# Assessment of a Robotic Walker in Older Adults With Parkinson's Disease in Daily Living Activities

**DOI:** 10.3389/fnbot.2021.742281

**Published:** 2021-12-14

**Authors:** Sergio D. Sierra M., Daniel E. Garcia A., Sophia Otálora, María Camila Arias-Castro, Alejandro Gómez-Rodas, Marcela Múnera, Carlos A. Cifuentes

**Affiliations:** ^1^Department of Biomedical Engineering, Colombian School of Engineering Julio Garavito, Bogotá, Colombia; ^2^Programa de Fisioterapia, Fundación Universitaria del Área Andina, Pereira, Colombia; ^3^Programa Ciencias del Deporte y la Recreación, Universidad Tecnológica de Pereira, Pereira, Colombia

**Keywords:** smart walker, Parkinson's disease, daily living activities, senior fitness, timed up and go, older adults

## Abstract

The constant growth of the population with mobility impairments, such as older adults and people suffering from neurological pathologies like Parkinson's disease (PD), has encouraged the development of multiple devices for gait assistance. Robotic walkers have emerged, improving physical stability and balance and providing cognitive aid in rehabilitation scenarios. Different studies evaluated human gait behavior with passive and active walkers to understand such rehabilitation processes. However, there is no evidence in the literature of studies with robotic walkers in daily living scenarios with older adults with Parkinson's disease. This study presents the assessment of the AGoRA Smart Walker using Ramps Tests and Timed Up and Go Test (TUGT). Ten older adults participated in the study, four had PD, and the remaining six had underlying conditions and fractures. Each of them underwent a physical assessment (i.e., Senior Fitness, hip, and knee strength tests) and then interacted with the AGoRA SW. Kinematic and physical interaction data were collected through the AGoRA walker's sensory interface. It was found that for lower limb strength tests, older adults with PD had increases of at least 15% in all parameters assessed. For the Sit to Stand Test, the Parkinson's group evidenced an increase of 23%, while for the Chair Sit and Reach Test (CSRT), this same group was only 0.04 m away from reaching the target. For the Ramp Up Test (RUT), the subjects had to make a greater effort, and significant differences (*p-value* = 0.04) were evidenced in the force they applied to the device. For the Ramp Down Test (RDT), the Parkinson's group exhibited a decrease in torque, and there were statistically significant differences (*p-value* = 0.01) due to the increase in the complexity of the task. In the Timed Up and Go Test (TUGT), the subjects presented significant differences in torque (*p-value* of 0.05) but not in force (*p-value* of 0.22) due to the effect of the admittance controller implemented in the study. Finally, the results suggested that the walker, represents a valuable tool for assisting people with gait motor deficits in tasks that demanded more physical effort adapting its behavior to the specific needs of each user.

## 1. Introduction

Human gait is a locomotion process in which the human body moves forward, alternating support in both lower limbs (Vaughan, 2003). Different musculoskeletal and neurological pathologies considerably affect balance and stability during this process (Mrozowski et al., 2007; Sammer et al., 2012; Pirker and Katzenschlager, 2017). In particular, stroke and spinal cord injuries are strongly related to locomotion disorders and significantly affect people's motor skills (Gheno et al., 2012; Cifuentes and Frizera, 2016). Parkinson's disease (PD) is another brain disorder that disrupts these capabilities (World Health Organization, 2006). The gradual decline of cognitive faculties (Nieuwboer et al., 2001; Buchman et al., 2011; Belghali et al., 2017) and the neuromuscular system in the older adults (Gheno et al., 2012; Poewe et al., 2017) are also associated with these pathologies. Besides, it is worth highlighting that PD is the second most common neuro-degenerative disorder affecting between 2 and 3% of the population aged 65 or older (Poewe et al., 2017).

The WHO estimates that the proportion of the population with mobility difficulties has been slowly and substantially rising, reaching 15% of the global population nowadays (World Health Organization, 2015). The United Nations also states that the world's population of older people will double in the next 3 decades, increasing from 9.3% in 2020 to 16% in 2050 (United Nations, 2020). Thus, mobility problems are common in older people and individuals with functional and cognitive disorders (Brown and Flood, 2013; Pedersen et al., 2014; Mikolajczyk et al., 2018). Several assistive devices have been developed to improve impaired locomotion abilities (Cifuentes and Múnera, 2022).

Concretely, mobility assistive devices help people overcome and compensate for physical disabilities by sustaining or improving their functioning and independence in clinical and daily situations (Van der Loos et al., 2016). One such device is traditional walkers with basic and low-cost mechanical systems, as well as, partial body weight support and stabilization. Nevertheless, such walkers compromise the balance and energy costs of the user, not to mention that fall prevention and overall safety are not very efficient (Neto et al., 2015; Sierra et al., 2022a). Another limitation of conventional devices is that they do not fully and correctly address cognitive and sensory assistance, which is of great importance for people with physical limitations (Mitzner et al., 2014; Jenkins and Draper, 2015; Geravand et al., 2016). Therefore, Smart Walkers (SWs) emerged, integrating robotic technologies to mitigate these drawbacks.

Smart Walkers are a potential tool for gait training and assistance due to their simple mechanical structures and multiple interaction interfaces (Scheidegger et al., 2019; Aristizabal-Aristizabal et al., 2022; Cifuentes and Múnera, 2022). The main functionalities of these devices include autonomous navigation systems (Papageorgiou et al., 2016), safety and obstacle avoidance modules (Sierra et al., 2018), biomechanical monitoring (Caetano et al., 2016; Alves et al., 2017; Sierra et al., 2021), user intention detection mechanisms (Lacey and Rodriguez-Losada, 2008), path-following modules (Sierra et al., 2022b), and people detection systems (Sierra et al., 2019). Also, these strategies provide a natural and safe interaction with the user in dynamic and complex environments (Neto et al., 2015). Therefore, they are often referred to as Human-Robot Interaction Interfaces (HRI) and Human-Robot-Environment Interaction Interfaces (HREI) (Sierra et al., 2019).

Several case studies report the effects of robotic walkers. As presented in Chugo et al. (2009), Jun et al. (2011), Yoon et al. (2012), and Werner et al. (2020), the efficacy and user satisfaction with sit-to-stand assistance systems provided by the walker is evaluated. These studies analyze how the device impacts the stability and balance of the subjects when the task is combined with a brief walk. Although relevant results are presented in terms of improved performance during the tests, there is no evidence of studies where the difficulty of the tests is increased with the aim of both physically and cognitively stimulating the user (i.e., more extended gait tasks and turns before the subject sits down). On the other hand, other studies have also been presented where the effect of the walker on the gait pattern of the subjects in scenarios that emulate daily activities (Wang et al., 2014; Lindemann et al., 2016, 2017; Costamagna et al., 2019; Mundt et al., 2019).

Studies involving subjects suffering from neurologica diseases have been also reported (Martins et al., 2015; Bayon et al., 2016; Moreira et al., 2019). Regarding subjects with Parkinson's disease, some studies focused on interaction strategies to improve the experience during the task (Mou et al., 2012; Zhang et al., 2018). These kinds of studies evaluated the level of assistance provided by the device and how it influenced the speed, cadence, and stability of the users (Cubo et al., 2003; Kegelmeyer et al., 2013; Wu et al., 2020). However, exploration of the kinematic effects of robotic walkers in this population remains scarce. In particular, there is insufficient evidence of the validation of these devices in more dynamic environments that emulate daily tasks. Furthermore, at the time of the writing of this manuscript, no studies compare the performance of people with PD with other focal groups with different physical and neurological conditions.

In this sense, the main contribution of this study is the comparison of the kinematic performance of a group of older adults with PD vs. a group of older adults with metabolic diseases, joint diseases, and fractures. This assessment was conducted when the older adults were interacting with the AGoRA SW in tests such as Up Ramps, Down Ramps, and Timed Up and Go. Besides, it presents and analyzes how the robotic device helps to compensate for the limitation of the subjects in the tasks that demanded more physical effort. Additionally, this study analyzes how the physical conditions of the subjects and the interaction strategy of the walker influence the results obtained. For this purpose, the Senior Fitness Test (SFT), lower limb strength tests, and a human-robot interaction strategy were necessary for this study.

## 2. Materials and Methods

This section describes the robotic platform used during the study and the interaction strategy proposed to provide an appropriate level of assistance to the user. Moreover, this part details the tests performed and the experimental setup, and the data collected in each of them.

### 2.1. Robotic Platform Description

**Figure 1:**
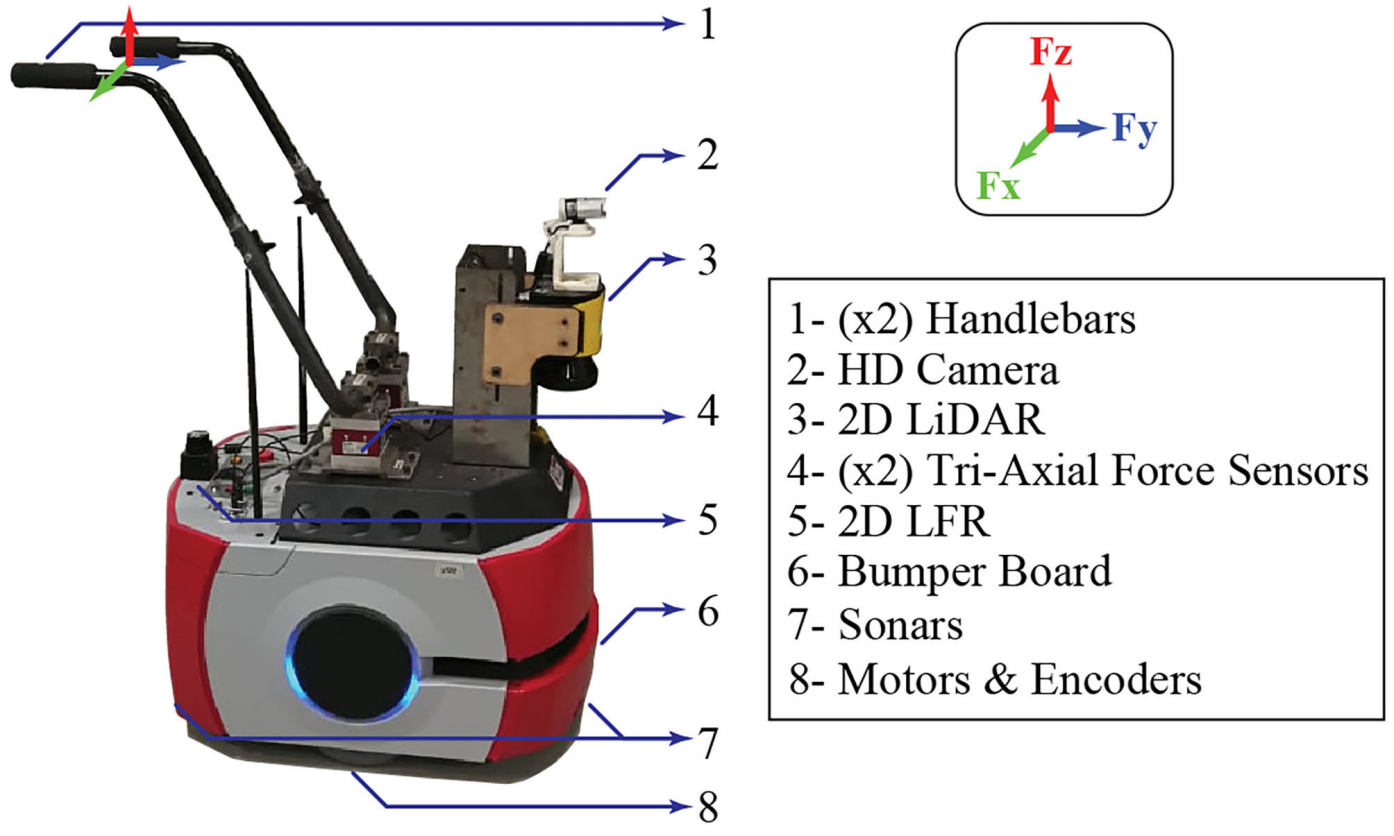
AGoRA Smart Walker (SW) description.

The Pioneer LX research platform (Omron Adept Technologies, Pleasanton, CA, USA) referred to as the AGoRA SW was used for this study. As presented in Sierra et al. (2019), this device was adapted to emulate the structural frame of a conventional assistive walker by attaching two forearm support handlebars to the main deck of the platform (refer to Figure 1).

This platform equips an onboard computer running a Linux operating system distribution compatible with the Robotic Operating System (ROS) framework. Moreover, features different sensors, actuators, and processing units such as (1) two motorized wheels and two caster wheels that provide propulsion and stability to the walker; (2) two encoders and an inertial measurement unit (IMU) to estimate the position and orientation of the device; (3) a 2D light detection and ranging (LiDAR) sensor (S300 Expert, SICK, Waldkirch, Germany) to sense the environment and detect obstacles; (4) two ultrasonic plates to detect objects at low hight; (5) two triaxial load cells (MTA400, FUTEK, Irvine, CA, USA) to estimate the user's navigation commands; (6) an HD camera (LifeCam Studio, Microsoft, Redmond, WA, USA) for human detection; and (7) a 2D laser rangefinder (Hokuyo URG-04LX-UG01, Osaka, Japan) to estimate the user's gait parameters (Sierra et al., 2019).

### 2.2. System Operation

**Figure 2:**
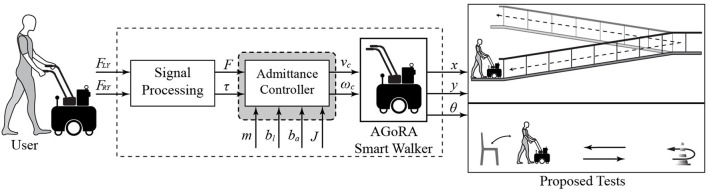
Description of the system architecture implemented in this study.

In this study, the architecture described in Figure 2 was implemented. The overall system is composed of two main modules: (1) a signal processing module, which is in charge of filtering the signals from the force sensors and generating the corresponding resulting forces and torques, and (2) an admittance controller, which converts the user's movement intention to speed commands to provide an appropriate level of assistance when they interact with the SW.

#### 2.2.1. Signal Processing Module

As presented in (Sierra et al., 2019) between the SW force sensors and the user's handlebar points, there is a vertical misalignment (refer to Figure 1). Supported by previous studies (Sierra et al., 2019, 2021), this implies that the resulting forces along the *y-axis* and *z-axis* read by the sensors will combine the forces along the *y-axis* and *z-axis* at the support points. However, it is possible to estimate that the forces along the *y-axis* provide essential information related to the user's motion intention. Similarly, the forces along the *z-axis* are a directly proportional estimation of the user's support on the device. At the same time, the forces along the *x-axis* are discarded, since they do not provide relevant information (Sierra et al., 2019). These signals are contaminated with some noise sources, related to the natural oscillatory pattern of gait (Brodie et al., 2015) and vibrations associated with irregularities in the floor (Sierra et al., 2019), which implies that these signals require additional filtering and conditioning treatment to remove such artifacts.

Hence, the same filtering strategy presented in Sierra et al. (2019) is implemented in this study, which mainly consists of four steps: (1) averaging the force signals along the *z-axis*, which contain information related to the oscillatory movements of the user's trunks, then (2) a band-pass filter is applied to remove all high-frequency components (1-2 *Hz* cutoff frequencies), then (3) the resulting signal cadence is estimated, thanks to the weighted fourier linear combiner filter (Frizera et al., 2010), and finally (4) the oscillatory components of the forces along the *y-axis* of each sensor are removed by introducing the cadence into a fourier linear combiner filter (Frizera Neto et al., 2010).

After performing this process, it is possible to estimate the force (F) and the resulting torque (t) and, thus, have an indicator that provides relevant information of the physical interaction between the SW and the user. Equations (1) and (2) describe how the signals are finally obtained:

(1) $$\begin{array}{r} \vec{F}={\vec{F^{\prime }}}_{LY}+{\vec{F^{\prime }}}_{RY}, \end{array}$$

(2) $$\begin{array}{r} \vec{\tau }=\left({\vec{F^{\prime }}}_{LY}-{\vec{F^{\prime }}}_{RY}\right)*\frac{d}{2}. \end{array}$$

${\vec{F^{\prime }}}_{LY}$ and ${\vec{F^{\prime }}}_{RY}$ are the filtered force signals from both handlebars, and *d* is the separation between the load cells of the device, which for this case is 0.3 m. It is essential to highlight that the force provides information about the user's intention when starting to walk, while the torque supplies information about the user's intention when turning.

#### 2.2.2. Interaction Strategy

Admittance controllers, widely used in SWs, are dynamic models that allow the robotic device to respond efficiently to the user's motion intentions (Jiménez et al., 2019). This sort of strategy allows to virtually modify the mechanical impedance of the walker, allowing to emulate different levels of assistance (Jiménez et al., 2019; Sierra et al., 2021, 2022b). With these controllers, it is possible to generate speed commands according to the user's exerted force and torque. Depending on the controller's constants, the SW can resemble lightweight device or a heavy device (Sierra et al., 2021). Thus, the purpose of these strategies is to provide users with feelings of easiness and naturalness during physical interaction with the robotic walker.

This study implements two admittance controllers to generate linear and angular velocities from the force and torque signals applied by the user on the handlebars. The controllers model the SW as a first-order *mass-damper* system and the outputs are linear (*v*) and angular (ω) velocities, as described in Equations (3) and (4):

(3) $$\begin{array}{r} L\left(s\right)=\frac{v\left(s\right)}{F\left(s\right)}=\frac{\frac{1}{m}}{s+\frac{b_{l}}{m}}, \end{array}$$

(4) $$\begin{array}{r} A\left(s\right)=\frac{\omega \left(s\right)}{\tau \left(s\right)}=\frac{\frac{1}{J}}{s+\frac{b_{a}}{J}}, \end{array}$$

where *m* is the walker's virtual mass, *J* is the virtual moment of inertia of the walker, and *b*_*l*_ and *b*_*a*_ are damping constants. These equations describe the transfer function of each controller. *L*(*s*) stands for Linear System, and *A*(*s*) stands for Angular System. It was necessary to adjust the values of the controller parameters to achieve an appropriate SW behavior. For this purpose, the virtual mass (*m*), inertia (*J*), and damping constants (*b*_*l*_ and *b*_*a*_) were adjusted after several experimental tests with healthy subjects (Sierra et al., 2021). In particular, the following values were used: *m*  = 0.5 *kg*, *b*_*l*_  = 4 *N*.*s*/*m*, *J*  = 2.1 *kg*.*m*^2^/*rad*, and *b*_*a*_ = 2 *N*.*m*.*s*/*rad*.

Regarding mass and inertia, low values were required since the AGoRA walker is a heavy robotic platform (70.2 kg). The inertia value was designed to be at least two times the virtual mass to ensure balance and stability during walking.

### 2.3. Experimental Protocol

This section describes the implemented experimental protocol to assess the interaction between the users and the AGoRA SW during the proposed tests. Additionally, it presents the physical assessment tests that were performed on each of them.

#### 2.3.1. Session Environment

This study took place at the Innovation and Technological Development Center (ITDC) of the Technological University of Pereira. The tests were performed jointly with physiotherapists and professors from the Areandina University Foundation.

#### 2.3.2. Participant Recruitment

- Inclusion criteria: Adults over 65 years of age who present some type of physical or cognitive condition that will significantly affect their gait cycle.
- Exclusion criteria: Subjects who did not present pathologies associated with alterations of normal gait parameters were excluded from the study.

A group of subjects that were actively attending a rehabilitation program was formally recruited to participate in the clinical study. The ethics committee previously approved the study, and all participants read and signed the written informed consent. The group was conformed by ten subjects (5 men, 5 women, 69.5 ± 8.44 y.o, 1.61 ± 0.08 m, 66.35 ± 14.93 kg), 4 of them had PD and, the remaining 6 had metabolic and joint diseases, and some had previous fractures. Table 1 describes the demographic data of the participants.

**Table 1:** Summary of demographic data of the volunteers who participated in the clinical study.

**Subject**	**Group**	**Gender**	**Age**	**Weight [kg]**	**Height [m]**	**IMC**	**Pathology**
1	PK	Male	70	83	1.73	28	Parkinson
2		Male	71	59	1.66	22	Parkinson
3		Male	70	82	1.70	28	Parkinson
4		Female	70	57	1.63	22	Parkinson
5	MJF	Female	68	74	1.55	31	Arterial Hypertension Osteoarthritis
6		Female	67	57	1.58	23	Hypothyroidism Osteoporosis
7		Female	71	61	1.53	26	High Blood Pressure Osteoporosis
8		Male	72	70	1.64	26	Epicondylitis Osteoporosis
9		Female	67	50	1.47	23	Triple Ankle Fracture
10		Male	69	70	1.62	27	Tibial Plate Fracture

*Parkinson group (PK) is the group suffering from PD and, MJF is the group of older adults with metabolic, joint diseases, and fractures*.

#### 2.3.3. Session Procedure

Before starting the tests, participants had to fill out an informed consent form to ensure that they had voluntarily expressed their intention to participate in the research. Participants only had to attend one session (i.e., a total of 10 sessions were conducted.), which was divided into two stages: (1) physical validation tests and (2) tests with the robotic walker. The participant had 1 h of rest between each of these to prevent them from becoming fatigued or experiencing any kind of muscular load.

The first stage aimed at determining the physical condition of the subjects. Several tests evaluated their levels of resistance, strength, and flexibility. Specifically, a digital dynamometer (microFET2, Hoggan Scientific, USA) was used to measure the force exerted by both the hip and the knee. Measurements with this type of device have been shown to be valid and reliable (Kawaguchi and Babcock, 2010). In the case of the hip, for the assessment of flexor and extensor strength, participants were placed supine with the hip flexed at approximately 90°. Abductor and adductor strength were measured while participants were lying on their side with 0° of hip flexion/extension. Additionally, for knee extensor and flexor strength, participants were required to be seated with approximately 90° hip and 90° knee flexion (Mintken et al., 2007; Stevens-Lapsley et al., 2010). Participants performed a series of maximal voluntary isometric contractions (MVIC) preceded by two submaximal warm-up contractions. All participants received visual targets and solid verbal encouragement during each MVIC to assist in obtaining maximal effort. All MVICs were performed by allowing the patient to increase the force to maximal capacity gradually; the maximal effort was maintained for 3–5 s. Patients were allowed 30-s rest periods between repetitions.

Furthermore, the physical capabilities and functional skills of the participants were assessed using the SFT (Rikli and Jones, 2013; Hesseberg et al., 2015). The following tests were performed:

- Sit to Stand Test (SST): This test consisted of counting the number of times the participant stood up from a chair with his arms crossed on his chest for 30 s. The purpose of this test was to evaluate the strength and resistance of the subjects' legs.
- Arm Curl Test (ACT): This test consisted of counting how many times the participant managed to bend the forearm with a weight (5 pounds for women, 8 pounds for men) for 30 s. This test aimed to measure the strength and resistance of the subjects in their upper limbs.
- Chair Sit and Reach Test (CSRT): This test consisted of measuring the distance the participant was missing to reach the toe (minus score) or beyond the toe (plus score). While sitting on the edge of a chair, one leg should be bent and the foot flat on the floor, while the other leg was extended straight in front of the hip with the heel on the floor and the foot ankle 90°. The person leaned forward at the hip while sliding the hands along the extended leg (the position was held for 3 s). The purpose of this test was to evaluate the flexibility of the lower limbs of the subjects.
- Back Scratch Test (BST): This test consisted of measuring the distance between (or overlap of) the middle fingers behind the back when attempting to touch the middle fingers of both hands together behind the back. Such a test was intended to measure the overall range of movement of the subjects' shoulders.
- Six Minutes Walking Test (6MWT): This test is used as an endurance test and is often used as a general indicator of overall physical performance and mobility in older adults (Heerink et al., 2009). In this sense, the participants were instructed to walk over a flat hallway without running or jogging, and they were allowed to stop and rest during the test. Considering the test duration, a walking circuit was used, where the subject had to make a U-turn every 30 m. For this test, it was necessary to instrument the user with the G-WALK sensor (BTS G-Sensor, BTS Bioengineering, USA) to extract average speed and cadence parameters (BTS Bioengineering, 2019).

In both cases (i.e., lower limb strength tests and SFT), each participant had to perform three repetitions, except in the 6MWT, because of the time and distance covered by the user, only one repetition is sufficient (Rikli and Jones, 2013). These measurements were averaged and reported.

**Figure 3:**
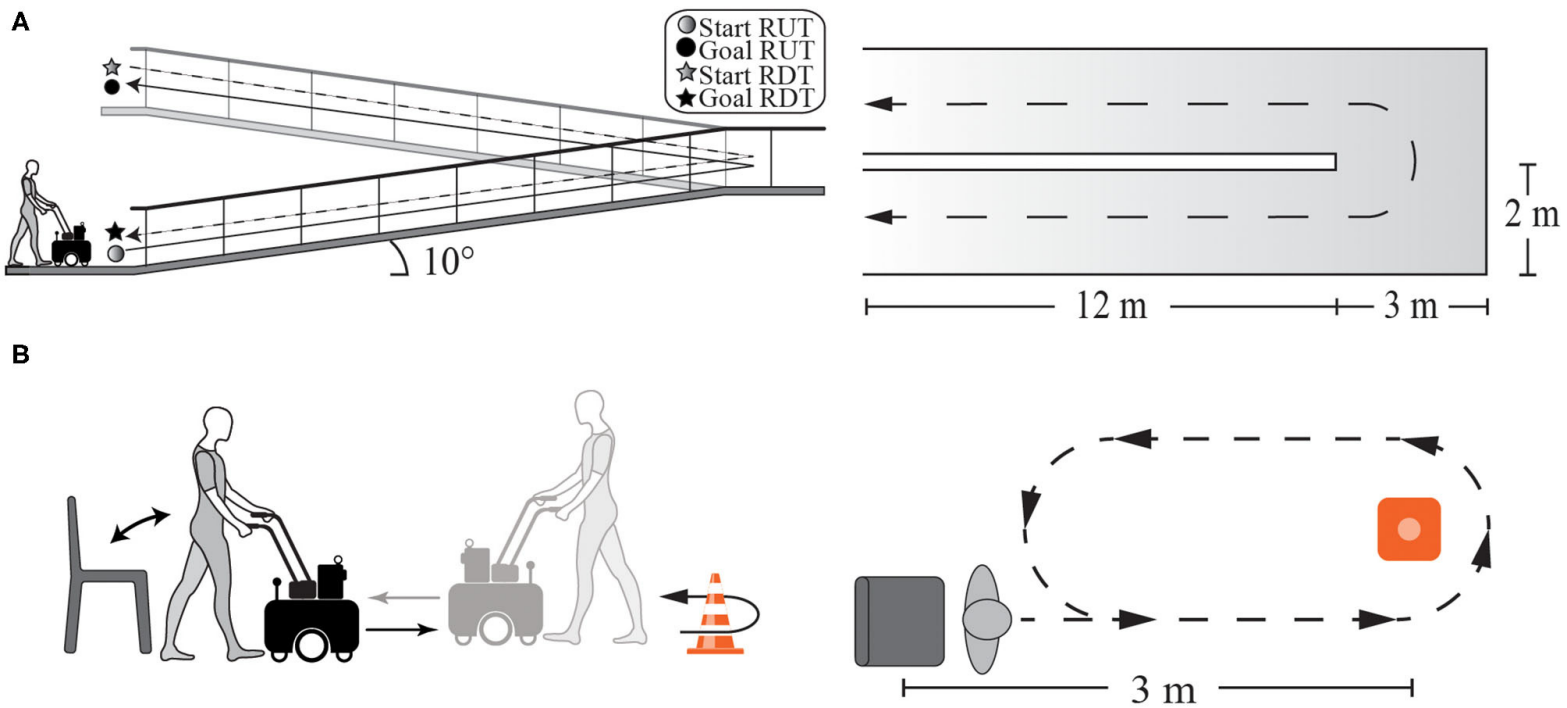
Illustration of experimental setups of the daily living activities part. **(A)** Ramps test setup. **(B)** Timed Up and Go test (TUGT) setup.

The second stage of the study compared the kinematic performance of both groups when they interacted with the AGoRA SW in everyday scenarios. The following tests were proposed:

- Ramps: The subjects were asked to walk up (RUT) and down (RDT) a ramp with the AGoRA Walker. Each user had to walk three times on the ramp-up and then three times on the ramp down (refer to Figure 3).
- Timed Up and Go test (TUGT): This is a clinical assessment test widely used to assess balance and walking ability in elderly populations (Heerink et al., 2009). A modified version of this test was used to be suitable during walker-assisted gait. Specifically, the subjects were asked to rise from a chair, walk at their usual pace a distance of 3 m, make a U-turn around a cone, walk back to the chair, and sit down (Heerink et al., 2009). Due to the use of the walker, users had to make a final turn before reaching the chair (refer to Figure 3).

Finally, data were acquired through the sensory interface of the AGoRA Walker. Specifically, the interaction force and torque, walker speeds, and trial duration were recorded. Moreover, to estimate additional parameters of the subject, the G-WALK sensor was used (BTS Bioengineering, 2019). This device supplied the most relevant parameters related to each trial, such as the subject's speed, cadence, gait cycle duration, and the number of cycles.

**Figure 4:**
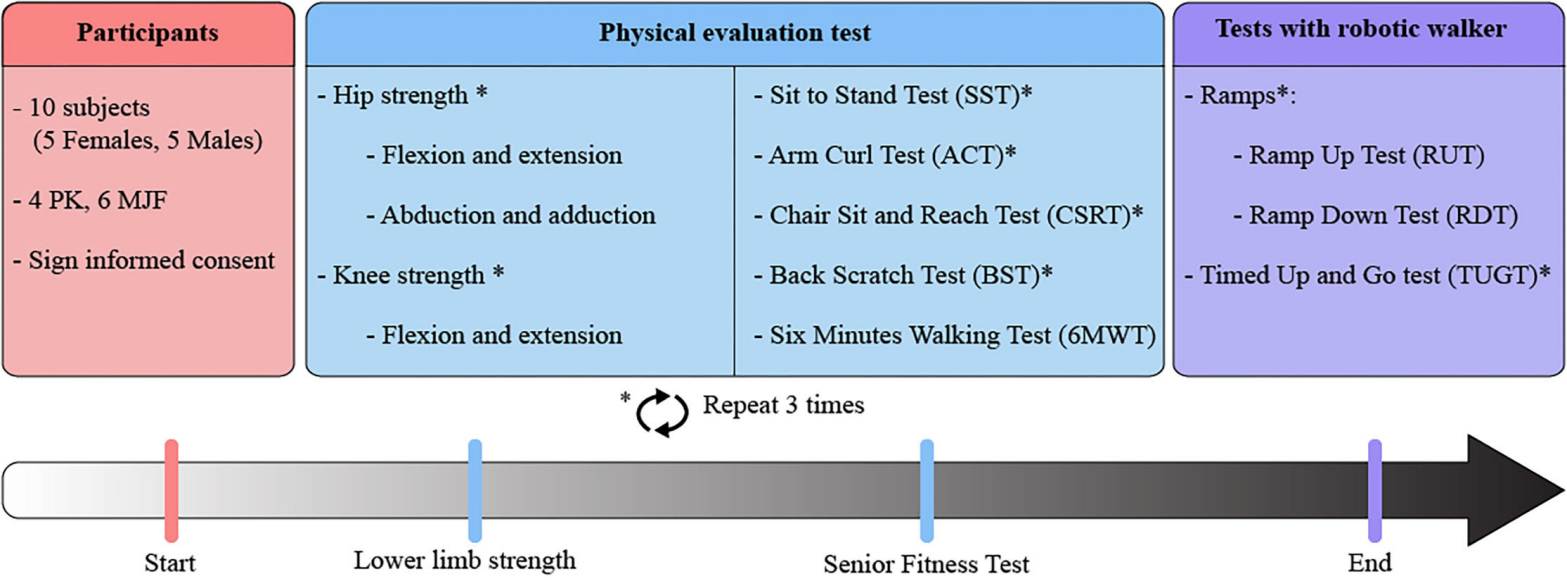
Summary of the protocol and experimental tests performed by the older adults to evaluate their performance within the study.

Figure 4 summarizes the procedure and tests that were performed to evaluate this study.

#### 2.3.4. Statistical Analysis

Descriptive statistics were used to report the results of the study. The significance level of all tests was set at 0.05. To determine the distribution of the information collected, the Shapiro-Wilk normality test was performed. To evaluate statistically significant differences, two types of tests were performed. In the case of parametric data, the non-paired *t*-test was performed. Regarding non-parametric data, Mann-Whitney tests were performed.

### 2.4. Ethics Statement

The University Research Ethics Committee approved this experimental protocol. Participants were informed of the scope and purpose of the experiment, and as explained above, their written informed consent was obtained before the study. Additionally, participants were free to leave the study whenever they chose to do so.

## 3. Results

Five hundred forty-six trials divided into 10 sessions were performed. Kinematic and interaction parameters were measured, such as users' gait spatio-temporal parameters, the interaction force and torque, trial average duration, and walking distance. This section describes the results obtained during the study.

### 3.1. Physical Condition Assessment Results

The data provided by the digital dynamometer were used to estimate the condition of the participants' lower limbs. Table 2 summarizes the strength values for the hip and knee of each group (i.e., PK and MJF). The dominant side of both groups was the right side, allowing a direct comparison between them.

**Table 2:** Force data obtained from the lower limb strength of the participants.

**Join**	**Parameter**	**Side**	**PK**	**MJF**	***p-value***
Hip	Flexion [N]	Right	198.10 ± 20.64^*^	147.13 ± 27.73^*^	**0.02**
		Left	166.94 ± 67.34^*^	142.27 ± 29.19^*^	**0.04**
	Extension [N]	Right	139.60 ± 19.05^*^	128.40 ± 37.76	**0.01**
		Left	127.98 ± 24.35^*^	126.62 ± 53.41	**0.05**
	Abduction [N]	Right	127.89 ± 27.21^*^	108.03 ± 18.22^*^	**<0.01**
		Left	126.33 ± 17.77^*^	98.69 ± 23.85^*^	**0.04**
	Adduction [N]	Right	118.20 ± 2.71^*^	95.81 ± 45.28	**<0.01**
		Left	117.46 ± 29.41^*^	92.94 ± 40.11^*^	**<0.01**
Knee	Flexion [N]	Right	147.71 ± 25.63^*^	118.81 ± 29.96^*^	**0.03**
		Left	147.03 ± 25.63^*^	95.62 ± 31.12^*^	**0.05**
	Extension [N]	Right	185.83 ± 13.38^*^	166.53 ± 93.40^*^	**0.04**
		Left	185.44 ± 41.06^*^	155.83 ± 74.65^*^	**<0.01**

*Asterisks indicate the data have a normal distribution. P-values in bold indicate significant differences between groups*.

Regarding hip flexion and extension, Table 2 shows how the group of older adults with Parkinson presented better results. The MJF group presented a reduction of 25.7% (right limb) and 17.34% (left limb) compared to the PK group during flexion tests. Besides, significant differences were found between both groups (*p-value* of 0.02). In extension, the strength values in both the right and left were more remarkable for the PK group. Although this parameter presented significant differences, there is not a great discrepancy in the strength measurements for the left hemisphere since the Parkinson group presented a mean of 127.98 N and the group of older adults presented a mean of 126.62 N.

Concerning the movements of abduction and adduction, both parameters presented statistically significant differences. On the right side, the PK group showed an increase compared to the MJF group of 18.38% in abduction, while in adduction, such an increase was 23.37%. The difference between the groups was considerably more significant for the left side since, for both parameters, the increase exceeded 25%.

Table 2 presents the results obtained in the flexion and extension measurements of the participants. The MJF group presented a mean value of 118.81 N vs. a 137.71 N by the PK group in flexion for the right hemisphere, representing an increase of 15.90%. This behavior did not change for the left side, as the PK group exhibited a 43.31% increase. Regarding joint extension, the MJF group presented in each case lower results. Also, significant differences were found in the two parameters.

On the other hand, Table 3 presents the results obtained from the SFTs. It can be observed that for the ACT and BST, which evaluated the upper body condition of the subjects, there were no significant differences (*p-values* of 0.74 and 0.06, respectively). This behavior was maintained for the 6MWT (*p-value* of 0.53), which was used as a general indicator of the older adults' overall physical performance and mobility. The remaining tests, which evaluated the lower body condition of the subjects, presented statistically significant differences.

**Table 3:** Senior Fitness Test (SFT) results.

**Test**	**PK**	**MJF**	***p-value***
SST [repetitions]	11 ± 1.63^*^	10 ± 2.61^*^	**0.02**
ACT [repetitions]	13 ± 2.16^*^	14 ± 4.38^*^	0.74
CSRT [m]	−0.12 ± 0.09^*^	0.04 ± 0.02^*^	**0.03**
BST [m]	−0.20 ± 0.04^*^	0.09 ± 0.06^*^	0.06
6MWT [m]	395.4 ± 190.28^*^	383.2 ± 157.36^*^	0.53

*P-values in bold indicate significant differences between groups. Asterisks indicate a normal distribution of the data*.

**Figure 5:**
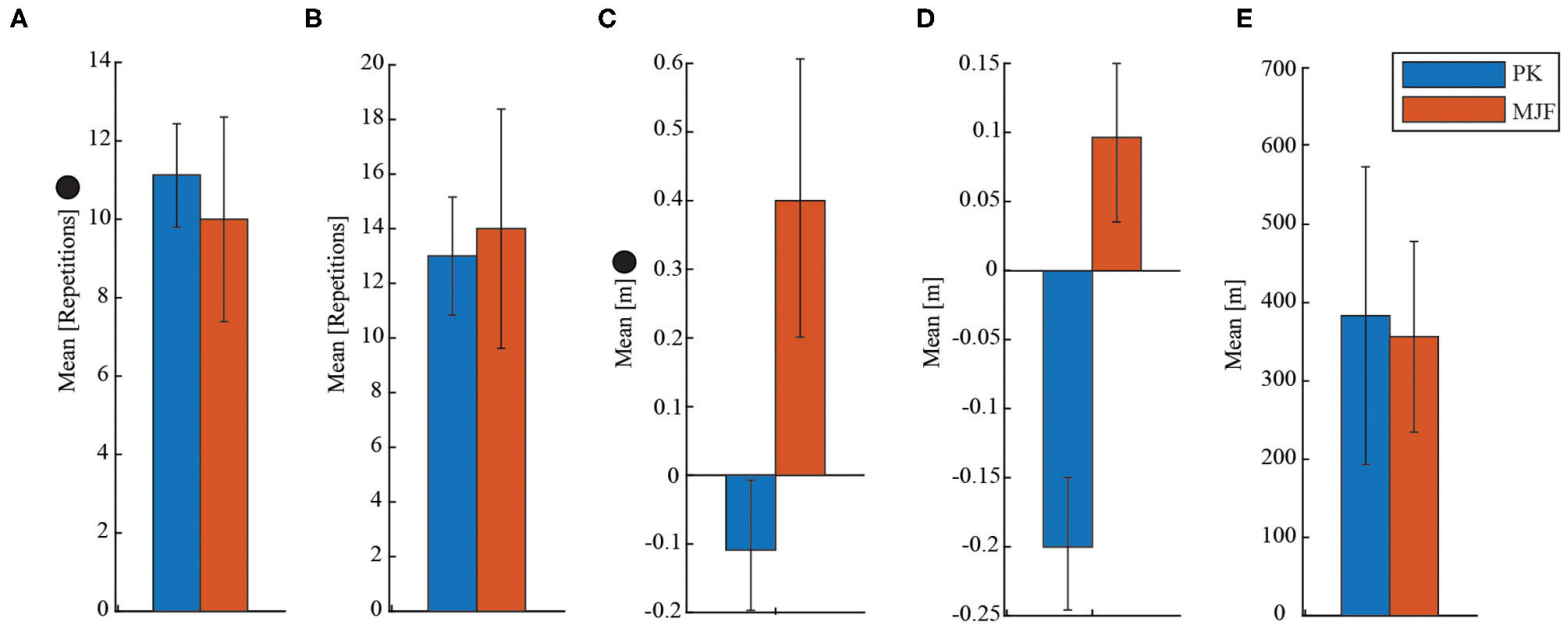
Illustration of the results obtained for the SFT. • indicates that there were significant differences between the groups evaluated. **(A)** Sit to Stand Test (SST). **(B)** Arm Curl Test (ACT). **(C)** Chair Sit and Reach Test (CSRT). **(D)** Back Scratch Test (BST). **(E)** Six Minutes Walking Test (6MWT).

For SST, although there were statistically significant differences, the PK group was only one repetition above the MJF group. Likewise, the group with metabolic and joint diseases showed greater flexibility in the lower limb (CSRT, refer to Table 3) as they were 0.04 m ahead of the toe, while the PK group was 0.12 m short of the target. Also, there were significant differences between the two groups. In addition to the above, to illustrate the behavior of these results, Figure 5 shows their distribution in a bar chart with the SD.

### 3.2. Kinematic and Physical Interaction Results

Several indicators were estimated using the data collected by the force sensors of the walker and the G-WALK sensor. To characterize the users' gait, parameters such as gait speed, cadence, average gait cycle duration, and the number of gait cycles were calculated. To evaluate the physical interaction between the participant and the walker, the mean force in the *y-axis* and *z-axis* were estimated, as well as, the maximum values.

Table 4 shows the kinematic and physical interaction data in the RUT, exceptthe parameters related to the torque in the *z-axis*, the remaining ones presented statistically significant differences. In terms of user and device speed, the PK group exhibited better results. Nevertheless, for cadence, cycle time, number of cycles, test duration, and the force impressed to the walker on the axis, the MJF group presented higher values supporting the previous results. However, for the *y-axis* force, very similar results were obtained.

**Table 4:** Results obtained for the Ramp Up Test (RUT).

**RUT**	**PK**	**MJF**	***p-value***
SW speed [m/s]	0.71 ± 0.07	0.61 ± 0.07	**<0.01**
User speed [m/s]	0.70 ± 0.07^*^	0.61 ± 0.07^*^	**<0.01**
Cadence [steps/min]	86.46 ± 3.28^*^	83.10 ± 5.28^*^	**0.02**
Cycle Duration [s]	1.39 ± 0.05^*^	1.45 ± 0.09^*^	**0.04**
No. Cycles	35.36 ± 4.36^*^	39.10 ± 4.77^*^	**0.01**
Trial Duration [s]	49.01 ± 5.34	56.70 ± 7.24^*^	**<0.01**
Max frc_y [N]	7.35 ± 0.88^*^	9.70 ± 1.05	**0.04**
Mean frc_y [N]	2.40 ± 0.28^*^	2.76 ± 0.28^*^	**0.04**
Max. trq_z [N.m]	4.44 ± 0.93^*^	4.86 ± 0.99	0.09
Mean trq_z [N.m]	0.01 ± 0.48^*^	0.03 ± 0.29^*^	0.27

*The p-values in bold indicate significant differences between the two groups, whereas the asterisks indicate that the data have a normal distribution*.

Further, as shown in Table 5, the kinematic and physical interaction parameters presented significant differences in their totality. For the speed of the walker and the user, the PK group showed better results. For the parameters related to the subject's cycles, cadence, and trial duration, the MJF group presented values below the remaining older adults. However, for user-generated force and torque, the MJF group exhibited considerably higher results.

**Table 5:** Results obtained in the Ramp Down Test (RDT).

**RDT**	**PK**	**MJF**	***p-value***
SW speed [m/s]	0.62 ± 0.07	0.56 ± 0.10	**0.03**
User speed [m/s]	0.62 ± 0.07^*^	0.56 ± 0.10	**0.05**
Cadence [steps/min]	104.67 ± 6.64^*^	96.78 ± 11.09^*^	**<0.01**
Cycle Duration [s]	1.15 ± 0.07^*^	1.35 ± 0.13^*^	**<0.01**
No. Cycles	45.24 ± 8.77^*^	48.77 ± 9.84	**<0.01**
Trial Duration [s]	51.62 ± 6.89^*^	60.13 ± 19.05	**0.04**
Max frc_y [N]	6.66 ± 0.65^*^	9.09 ± 0.83^*^	**0.02**
Mean frc_y [N]	2.19 ± 0.21^*^	4.73 ± 0.40^*^	**<0.01**
Max. trq_z [N.m]	7.20 ± 0.94^*^	9.05 ± 0.92^*^	**0.03**
Mean trq_z [N.m]	1.55 ± 0.14	1.91 ± 0.28	**<0.01**

*Asterisks indicate that the data followed a normal distribution. Bolded p-values indicate that there were statistically significant differences between groups*.

Regarding the TUGT, Table 6 summarizes the obtained results. Due to the nature of the test, since the user's speed is measured even before the user starts to move forward with the walker (i.e., in the standing and sitting stage), the parameters related to these speeds presented slight differences. In contrast to the previous tests, the cadence of the MJF group is higher than that of the PK group. Thus, the cycle length and the number of cycles of the group with PD are also higher. However, the PK group managed to finish the test in less time (32.33 s) than the other group (42.50 s). On the other hand, for the *y-axis* strength, both groups presented very similar results, adding that there were no significant differences. For the *z-axis* torque, which provides information on the user's support on the device, the PK group presented lower values.

**Table 6:** Results obtained for the Timed Up and Go Test (TUGT).

**TUGT**	**PK**	**MJF**	***p-value***
SW speed [m/s]	0.36 ± 0.09	0.32 ± 0.06	**<0.01**
User speed [m/s]	0.32 ± 0.08	0.29 ± 0.06	**<0.01**
Cadence [steps/min]	105.72 ± 13.87^*^	110.61 ± 29.58^*^	**0.04**
Cycle Duration [s]	1.17 ± 0.15^*^	1.13 ± 0.29	**0.05**
No. Cycles	38.82 ± 5.16^*^	36.59 ± 10.21^*^	**0.02**
Trial Duration [s]	32.33 ± 3.82^*^	42.50 ± 7.34^*^	**<0.01**
Max frc_y [N]	6.65 ± 0.67	6.68 ± 1.16^*^	0.74
Mean frc_y [N]	1.24 ± 0.27^*^	1.57 ± 0.31^*^	0.22
Max. trq_z [N.m]	5.51 ± 0.80^*^	6.07 ± 1.25	**0.03**
Mean trq_z [N.m]	0.27 ± 0.54	0.13 ± 0.85	**0.05**

*The bold parameters indicate significant differences between the two groups, whereas the asterisks indicate that the data have a normal distribution*.

**Figure 6:**
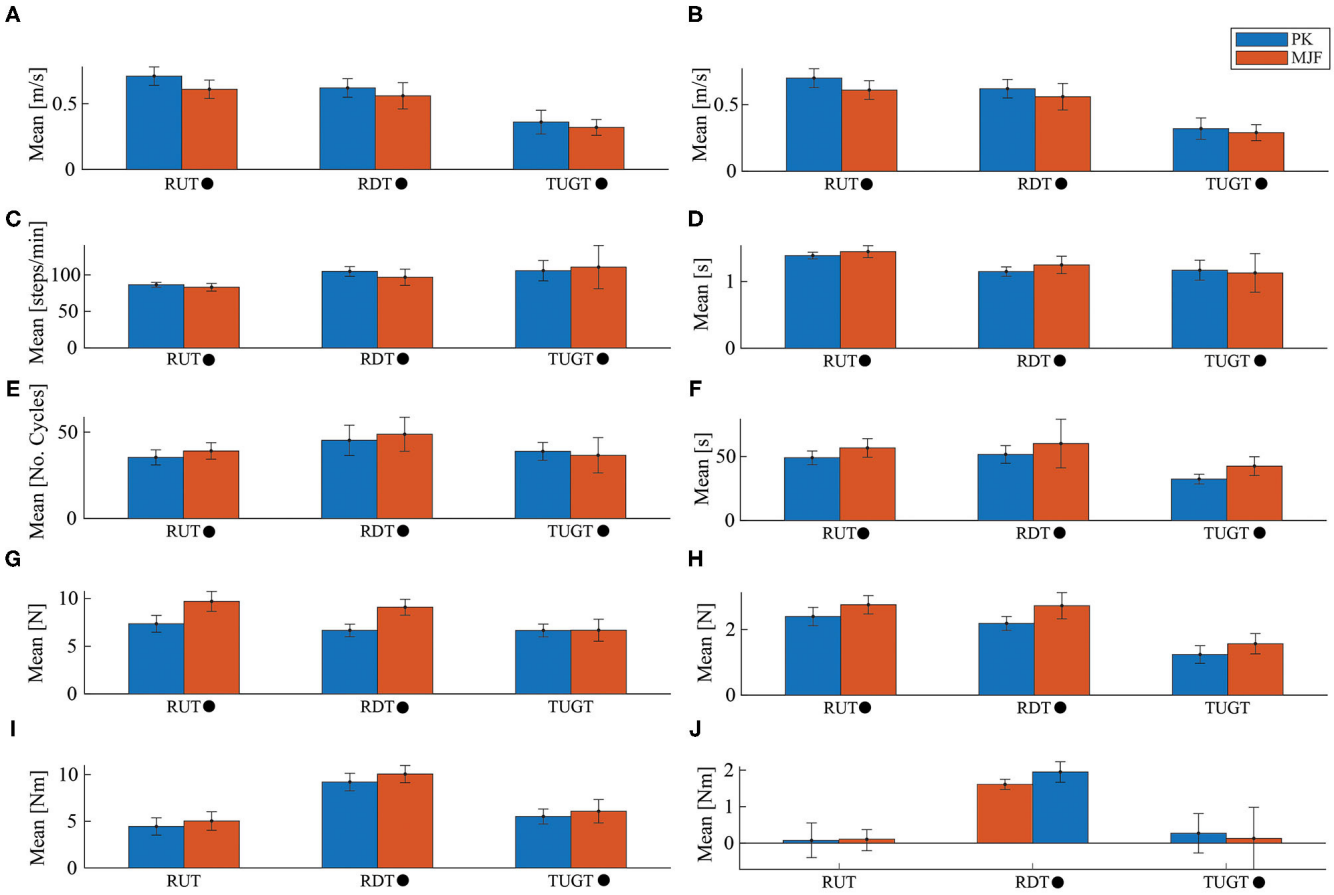
Illustration of the kinematic and physical interaction data obtained for the three tests. Where RUT is Ramp Up Test, RDT is Ramp Down Test, TUGT is Timed Up and Go Test, Parkinson group (PK) corresponds to the group of older adults with Parkinson's disease (PD) and MJF the group with metabolic and joint diseases and fractures. • indicates that there were significant differences between the groups evaluated. **(A)** User Speed. **(B)** SW Speed. **(C)** Cadence. **(D)** Cycle Duration. **(E)** No. Cycles. **(F)** Trial Duration. **(G)** Max frc_y. **(H)** Mean frc_y. **(I)** Max trq_z. **(J)** Mean trq_z.

**Figure 7:**
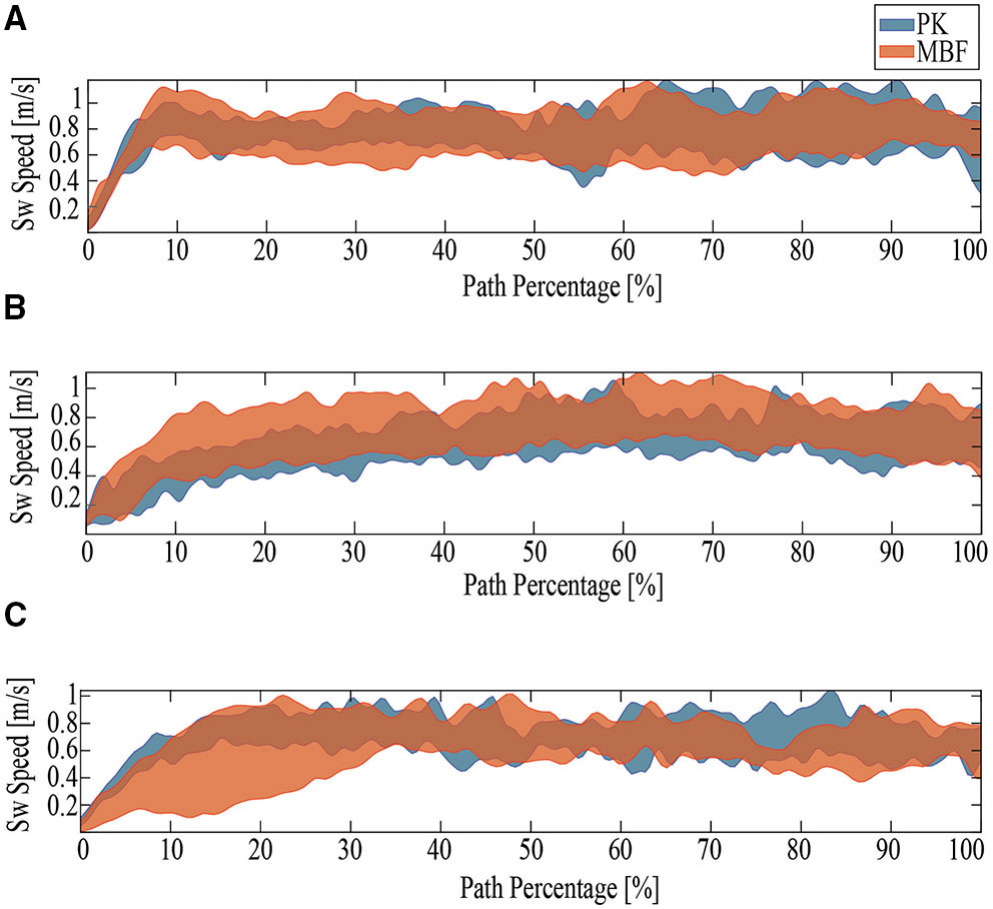
The behavior of the results obtained for walker speed. PK stands for older adults with Parkinson's disease and MJF stands for a group of older adults with base, joint and fracture diseases. **(A)** Ramp Up Test. **(B)** Ramp Down Test. **(C)** Timed Up and Go.

**Figure 8:**
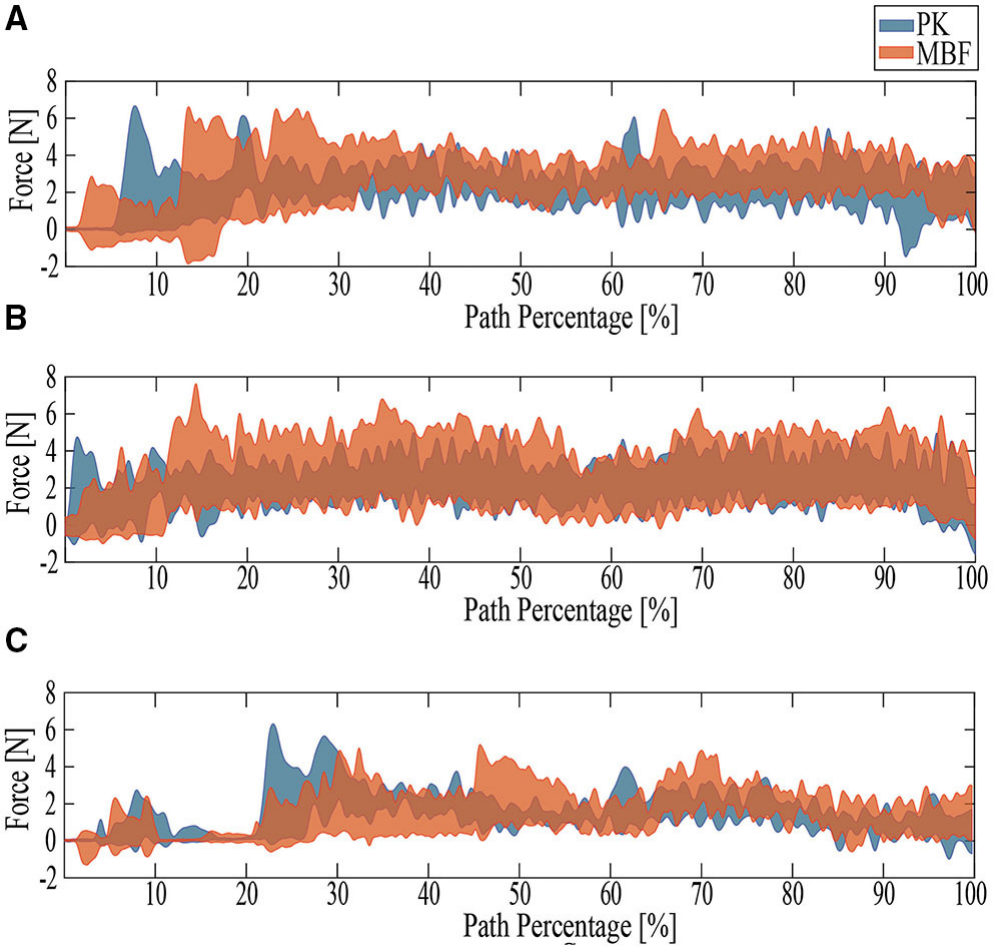
The behavior of the results obtained for force. PK stands for older adults with PD and MJF stands for a group of older adults with base, joint, and fracture diseases. **(A)** RUT. **(B)** RDT. **(C)** TUGT.

**Figure 9:**
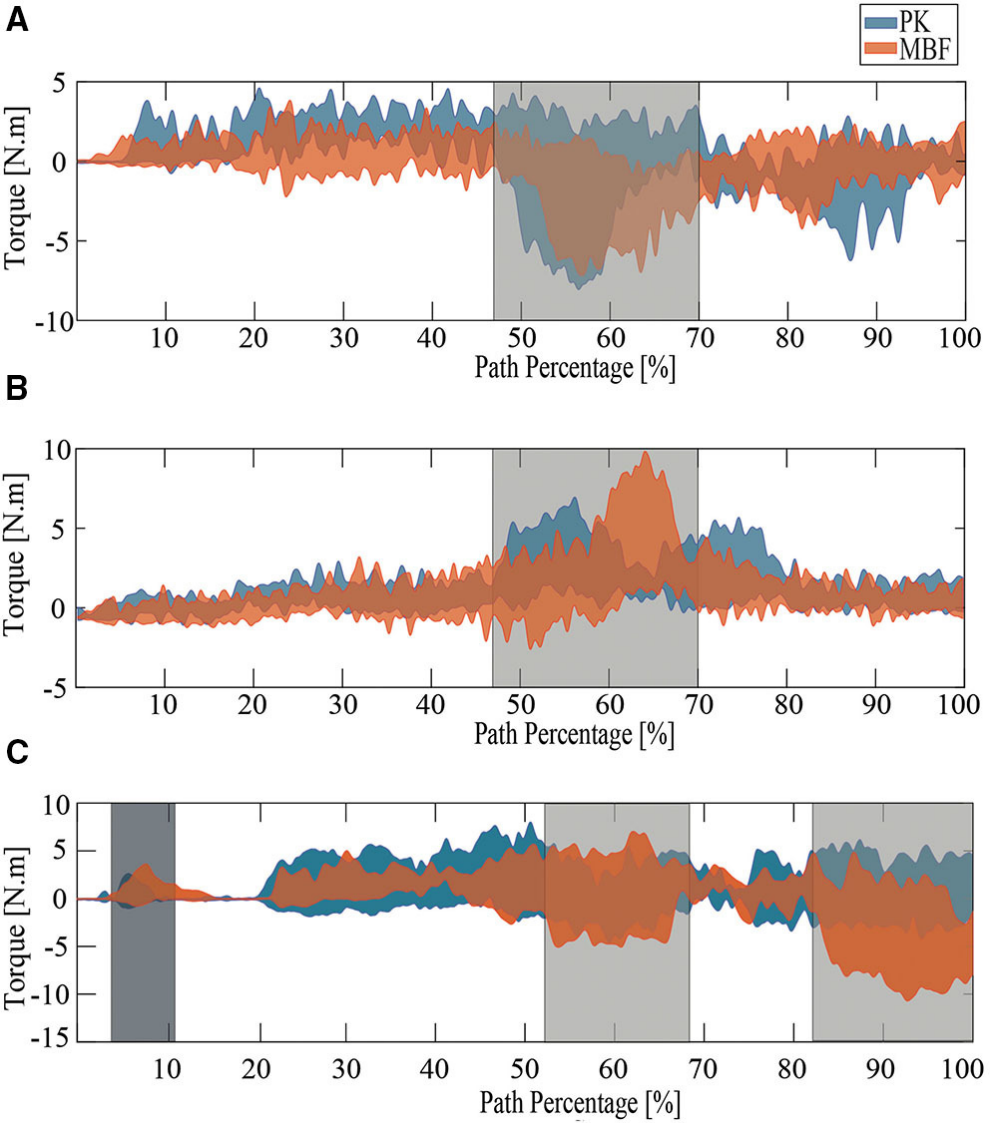
The behavior of the results obtained for torque. PK stands for older adults with PD and MJF stands for a group of older adults with base, joint, and fracture diseases. The areas highlighted in gray represent the times when the turns were presented and the area highlighted in black represents the time when the subjects were leaning on the device to stand up and initiate the test. **(A)** RUT. **(B)** RDT. **(C)** TUGT.

Figure 6 summarizes the parameters evaluated for the three proposed tests. Figures 7–9 show the walker speed, force, and torque behavior. For these illustrations, the data from all participants were averaged and the SD was used.

## 4. Discussion

In this study, there were no cases of misunderstanding of the behavior and operation of the AGoRA SW, and no cases of collisions were reported. Besides, it is essential to highlight that the sample size of this study is considered small. However, studies of similar samples have been reported with this type of devices, as well as, with older adults with Parkinson's and other diseases (Mou et al., 2012; Zhang et al., 2018; Mundt et al., 2019). According to the results presented in the previous section, it was observed that the physical condition of the subjects significantly influences the participants' performance using the device.

### 4.1. Physical Condition Assessment

The results shown in Table 2 indicate that the adults with PD were in better condition. For the hip strength, there was a considerable reduction in the MJF group compared to the PPK. Although PK group was expected to have lower hip strength (Inkster et al., 2003; Skinner et al., 2019), our results are supported by the fact that there are joint disorders (such as osteoarthritis and osteoporosis), which in advanced stages, affect and weaken the hip extensor and flexor muscles (Jerez-Mayorga et al., 2019). In Rydevik et al. (2010) and Judd et al. (2014), similar results were obtained, where a group of older adults with osteoarthritis exhibited a 10–25% deficit in hip muscle strength.

In terms of knee flexion and extension, the average deficit in this joint is less than that in the hip for the PK group. Moreover, the PK group flexion-extension outcomes were considerably higher than the MJF group. Such an increase was of more than 40% and statistically significant differences were found for the left limb flexion, mainly due to an older adult who had a fracture of the tibial plate in the MJF group. As presented in Gaston et al. (2005), when these types of fractures have not been given sufficient time for recovery, significant impairment of muscle movement and function can occur.

For the SFTs, the results presented in Table 3 were relevant to determining the participants' overall functional capacity, agility, dynamic balance, aerobic endurance, and upper and lower extremity muscular strength. The PK group exhibited better results in the Sit to Stand Test SST and significant differences were found (refer to Figure 5). These results are related to those previously obtained in the strength tests since the older adults in the MJF group showed a considerable reduction. Similarly, in Zijlstra et al. (2012) it is shown how Parkinson patients exhibit acceptable performance in this type of test.

Upper limb strength and endurance is another fitness parameter that was measured with the ACT. As shown in Figure 5, despite the slight increase in the number of repetitions in the test by MJF, there were no significant differences. Some older adults in this group who had osteoporosis and epicondylitis may have influenced the obtained results. Epicondylitis is an injury characterized by pain in the external aspect of the elbow, in the region of the epicondyle (Walz et al., 2010). Whereas, osteoporosis is a bone disease characterized by a decrease in the density of bone tissue and resulting in an exaggerated fragility of the bones (on Osteoporosis et al., 2001). In this sense, very similar results to those shown in Adamo et al. (2015) were obtained, indicating that participants with these pathologies show a significant deficit, compromising their physical abilities.

There was not much discrepancy in the mean values obtained for the CSRT results. However, significant differences were found in this parameter (refer to Figure 5). As in the previous test, this one presents values below normal. Additionally, these results are supported by the fact that older adults with Parkinson's present a direct relationship between the degree of stiffness their body experiences, generated by the disorder and the passing of years (Inkster et al., 2003).

In the opposite case, the MJF group presented better results for the BST, despite not showing significant differences (refer to Figure 5). Regarding the 6MWT the results were as expected. Even though, the PK group covered a slightly greater distance, there were no significant differences (refer to Figure 5). This last could be related to the fact that adults with PD cannot maintain a constant pace when making very long trips. As presented in Falvo and Earhart (2009), the Parkinson's group obtains similar results because of the subjects' impaired balance and predisposition to falls. For this reason and as presented in Mou et al. (2012), Zhang et al. (2018), and Wu et al. (2020), devices such as SWs can help reduce the risk factors for falls and help to maintain constant kinematic parameters such as speed and cadence of the participant in very long distances.

### 4.2. Kinematic and Physical Interaction

As for the Ramp Up Test (RUT), the PK group presented better results concerning user speed (refer to Figure 6), walker speed (refer to Figures 6, 7), and cadence (refer to Figure 6). In contrast, the MJF group obtained higher values in the cycle time, the number of cycles, and test duration (refer to Figure 6), meaning an inferior performance. This type of test involve muscles such as the glutes, hamstrings, and quadriceps (Lindemann et al., 2017), thus the flexion-extension results of the PK group support their better performance in the RUT. These results can also be seen in the force exerted by the users (refer to Figure 8), due to the considerable effort that the older adults in the MJF group had to make. This significant increase is also supported by the low performance in the hip and knee strength tests by this group. Given the weakened lower limbs in the MJF group, the participants felt the need to rely on the device to compensate for this deficit. However, there were no significant differences in the torque values as shown in Figures 6, 9, which is consistent with the nature of the test (i.e., the majority of the test with straight sections).

These results might classify the AGoRA SW as a potential tool for gait retraining and rehabilitation. Considering the participant's effort and the natural deterioration of their lower limbs, the implemented admittance controller could be adjusted to emulate a lighter or heavier platform according to the requirements of the subject.

Regarding the Ramp Down Test (RDT), the PK group also obtained better results. When comparing the behavior of the kinematic and physical interaction parameters during both ramp tests, similar results were observed in terms of user and walker speed (refer to Figures 6, 7). Slight changes were obtained in cadence, gait cycle time, and the number of cycles. These results are supported by the fact that uphill walking resulted in slower speeds because of the natural effort of the test. In addition, the peak force and torque (refer top Figures 8, 9, respectively) exerted by the users were considerably higher in the downhill ramp tests. Because the device exhibited higher moments of inertia during the downhill tests, subjects required greater efforts to perform the mid-ramp turn. These results are supported by the ACT (refer to Table 3), since adults with PD presented a 7.1% reduction in this test, indicating the natural deterioration of the lower limbs. For this reason, and unlike the previous test, there are significant differences in the torque generated in the z-axis. Similar results were obtained in Lindemann et al. (2017). In this study, they highlight how a SW reduces under-performance in subjects' gait and shows how the kinematic parameters (cadence, speed, cycles) are substantially reduced in the downhill tests.

Finally, during the TUGT, users were asked to lean on the device to stand out of the chair. To prevent the admittance controller from generating velocities, the device motors were remotely deactivated. This leaning event was observed as an initial spike in the force and torque signal. As seen in Figures 6, 8, the force signals did not exhibit many discrepancies, and there was no evidence of significant differences. Regarding the torque, it is essential to highlight that the MJF group showed higher values (refer to Figures 6, 9). These findings suggest that users saw in the intelligent walker a possibility to compensate for this deficit in the musculature (Judd et al., 2014). Moreover, compared to the previous tests, which required more physical effort from the subjects, the TUGT showed lower force (refer to Figure 6) and torque (Figure 6) values. This indicates the efficiency and usefulness of the admittance controller in emulating a lighter platform.

## 5. Conclusions and Future Work

This study presented the assessment of the AGoRA SW in daily life scenarios with two groups of older adults. One group had PD and the other group had underlying diseases, joint diseases, and fractures. Physical validation tests were performed and relevant results were found in the relationship between this condition and the performance in tests with the walker. One of the main findings of this study is related to the relationship between users' pathologies and their physical condition. As previously discussed, PD is a brain disorder that causes progressive cognitive and motor deterioration producing tremors, rigidity and difficulty in walking, balance, and coordination. However, it was found that pathologies such as osteoarthritis in advanced stages can further affect the physical condition of the subjects and their performance in activities of daily living.

Interesting results related to the kinematic and interaction parameters during the RUT were found. Although the RUT demands that the user has to apply a more significant effort even to the point of opposing the movement intention, it could induce muscle training during rehabilitation processes. In addition, this test could be complemented by adjusting the level of assistance of the AGoRA SW to meet the specific needs of each user. More resistive assistance levels induce slower gait patterns compared to studies reported in the literature. This could be interpreted as a safety strategy, as slower gait patterns could help users avoid collisions and stumbling while walking. In addition, the strength data collected during the RUT provided information on potential muscle training applications. Furthermore, as evidenced in the TUGT, the AGoRA SW can guarantee a natural and adequate interaction in scenarios where no significant effort is required from the users. This can be evidenced in the force values, as there were slight discrepancies but no significant differences. This indicates that despite the participant's physical condition and pathology, the AGoRA walker can assist efficiently.

One of the main limitations of this study is that it lacks EMG information that provides insights related to the participant's physical interaction and evolution with this technique in the different tests. As mentioned before, another limitation of this research is the sample size. However, this study is the first approach to comparing the performance of two groups of older adults with different physical and cognitive characteristics.

Finally, future study will be focused on evaluating at AGoRA SW with a bigger group of participants. The MJF group will include older adults with other pathologies that significantly affect their gait patterns. Besides, standardized scales will be included to determine the stage of the disease and how this may influence the results. Future studies will also include biomechanical analyses.

## Data Availability Statement

The datasets presented in this study can be found in online repositories. The names of the repository/repositories and accession number(s) can be found at: https://figshare.com/s/51dce22f5a718d759927.

## Ethics Statement

The studies involving human participants were reviewed and approved by Ethical Committee of the Colombian School of Engineering Julio Garavito. The patients/participants provided their written informed consent to participate in this study.

## Author Contributions

SS, DG, SO, MA-C, AG-R, MM, and CC contributed to the conception and design of the study. SS, MM, and CC conceptualized the study and designed the methodology. AG-R recruited the participants. SS, DG, and MA-C conducted the experimental trials. DG, MA-C, and SO performed data curation and processing. DG wrote the original manuscript. SS, AG-R, MM, and CC provided feedback on the manuscript. AG-R, MM, and CC supervised the study. MM and CC managed the funding resources. All authors have read and accepted the published version of the manuscript.

## Funding

This study was supported by the Ministry of Science, Technology, and Innovation of Colombia (Grant 801-2017) and (Grant 845-2020) and the Colombian School of Engineering Julio Garavito Funds.

## Conflict of Interest

The authors declare that the research was conducted in the absence of any commercial or financial relationships that could be construed as a potential conflict of interest.

## Publisher's Note

All claims expressed in this article are solely those of the authors and do not necessarily represent those of their affiliated organizations, or those of the publisher, the editors and the reviewers. Any product that may be evaluated in this article, or claim that may be made by its manufacturer, is not guaranteed or endorsed by the publisher.

## References

[B1] Adamo D. E. Talley S. A. Goldberg A. (2015). Age and task differences in functional fitness in older women: comparisons with senior fitness test normative and criterion-referenced data. J. Aging Phys. Act. 23, 47–54. 10.1123/JAPA.2012-0317 24412879

[B2] Alves J. Seabra E. Caetano I. Santos C. P. (2017). Overview of the asbgo++ smart walker, in 2017 IEEE 5th Portuguese Meeting on Bioengineering (ENBENG) (Coimbra: IEEE), 1–4.

[B3] Aristizabal-Aristizabal J. Ferro-Rugeles R. Lancheros-Vega M. Sierra M. S. D. Múnera M. Cifuentes C. A. (2022). Fundamentals for the Design of Smart Walkers, 1st Edn, Vol. 1 Cham: Springer International Publishing.

[B4] Bayon C. Ramírez O. Del Castillo M. D. Serrano J. I. Raya R. Belda-Lois J. M.. (2016). Cpwalker: robotic platform for gait rehabilitation in patients with cerebral palsy, in 2016 IEEE International Conference on Robotics and Automation (ICRA) (Stockholm: IEEE), 3736–3741.

[B5] Belghali M. Chastan N. Cignetti F. Davenne D. Decker L. M. (2017). Loss of gait control assessed by cognitive-motor dual-tasks: pros and cons in detecting people at risk of developing alzheimer's and parkinson's diseases. Geroscience 39, 305–329. 10.1007/s11357-017-9977-7 28551877 PMC5505895

[B6] Brodie M. A. D. Beijer T. R. Canning C. G. Lord S. R. (2015). Head and pelvis stride-to-stride oscillations in gait: validation and interpretation of measurements from wearable accelerometers. Physiol. Meas. 36, 857–872. 10.1088/0967-3334/36/5/857 25831990

[B7] Brown C. J. Flood K. L. (2013). Mobility limitation in the older patient: a clinical review. JAMA 310, 1168–1177. 10.1001/jama.2013.276566 24045741

[B8] BTS Bioengineering (2019). G-WALK.

[B9] Buchman A. S. Boyle P. A. Leurgans S. E. Barnes L. L. Bennett D. A. (2011). Cognitive function is associated with the development of mobility impairments in community-dwelling elders. Am. J. Geriatr. Psychiatry 19, 571–580. 10.1097/JGP.0b013e3181ef7a2e 21606900 PMC3101472

[B10] Caetano I. Alves J. Gonçalves J. Martins M. Santos C. P. (2016). Development of a biofeedback approach using body tracking with active depth sensor in asbgo smart walker, in 2016 International Conference on Autonomous Robot Systems and Competitions (ICARSC) (Bragança: IEEE), 241–246.

[B11] Chugo D. Asawa T. Kitamura T. Songmin J. Takase K. (2009). A motion control of a robotic walker for continuous assistance during standing, walking and seating operation, in 2009 IEEE/RSJ International Conference on Intelligent Robots and Systems (Bangkok: IEEE), 4487–4492.

[B12] Cifuentes C. A. Frizera A. (2016). Human-Robot Interaction Strategies for Walker-Assisted Locomotion, volume 115 of Springer Tracts in Advanced Robotics. Cham: Springer International Publishing.

[B13] Cifuentes C. A. Múnera M. (2022). Interfacing Humans and Robots for Gait Assistance and Rehabilitation 1st Edn, Vol. 1. Cham: Springer International Publishing.

[B14] Costamagna E. Thies S. B. Kenney L. P. Howard D. Lindemann U. Klenk J.. (2019). Objective measures of rollator user stability and device loading during different walking scenarios. PLoS ONE 14:e0210960. 10.1371/journal.pone.0210960 30699170 PMC6353162

[B15] Cubo E. Moore C. G. Leurgans S. Goetz C. G. (2003). Wheeled and standard walkers in parkinson's disease patients with gait freezing. Parkinsonism Relat Disord. 10, 9–14. 10.1016/S1353-8020(03)00060-9 14499200

[B16] Falvo M. J. Earhart G. M. (2009). Six-minute walk distance in persons with parkinson disease: a hierarchical regression model. Arch. Phys. Med. Rehabil. 90, 1004–1008. 10.1016/j.apmr.2008.12.018 19480877

[B17] Frizera Neto A. Gallego J. A. Rocon E. Pons J. L. Ceres R. (2010). Extraction of user's navigation commands from upper body force interaction in walker assisted gait. BioMed. Eng. Online. Vitória 9, 1–16. 10.1186/1475-925X-9-37 20687921 PMC2924341

[B18] Frizera A. Gallego J. Rocon de Lima E. Abellanas A. Pons J. Ceres R. (2010). Online cadence estimation through force interaction in walker assisted gait, in ISSNIP Biosignals and Biorobotics Conference 2010 (Vitoria), 1–5.

[B19] Gaston P. Will E. Keating J. (2005). Recovery of knee function following fracture of the tibial plateau. J. Bone Joint Surg. 87, 1233–1236. 10.1302/0301-620X.87B9.16276 16129749

[B20] Geravand M. Werner C. Hauer K. Peer A. (2016). An integrated decision making approach for adaptive shared control of mobility assistance robots. Int. J. Soc. Robot. 8, 631–648. 10.1007/s12369-016-0353-z

[B21] Gheno R. Cepparo J. M. Rosca C. E. Cotten A. (2012). Musculoskeletal disorders in the elderly. J. Clin. Imaging Sci. 2:39. 10.4103/2156-7514.99151 22919553 PMC3424705

[B22] Heerink M. Kröse B. Wielinga B. Evers V. (2009). Measuring the influence of social abilities on acceptance of an interface robot and a screen agent by elderly users, in People and Computers XXIII Celebrating People and Technology - Proceedings of HCI 2009, Cambridge, 430–439.

[B23] Hesseberg K. Bentzen H. Bergland A. (2015). Reliability of the senior fitness test in community-dwelling older people with cognitive impairment. Physiother. Res. Int. 20, 37–44. 10.1002/pri.1594 24925585

[B24] Inkster L. M. Eng J. J. MacIntyre D. L. Stoessl A. J. (2003). Leg muscle strength is reduced in Parkinson's disease and relates to the ability to rise from a chair. Mov. Disord. 18, 157–162. 10.1002/mds.10299 12539208 PMC3471985

[B25] Jenkins S. Draper H. (2015). Care, monitoring, and companionship: views on care robots from older people and their carers. Int. J. Soc. Robot. 7, 673–683. 10.1007/s12369-015-0322-y

[B26] Jerez-Mayorga D. Ríos L. J. C. Reyes A. Delgado-Floody P. Payer R. M. Requena I. M. G. (2019). Muscle quality index and isometric strength in older adults with hip osteoarthritis. PeerJ 7:e7471. 10.7717/peerj.7471 31410316 PMC6689221

[B27] Jiménez M. F. Monllor M. Frizera A. Bastos T. Roberti F. Carelli R. (2019). Admittance controller with spatial modulation for assisted locomotion using a smart walker. J. Intell. Robot. Syst. 94, 621–637. 10.1007/s10846-018-0854-0

[B28] Judd D. L. Thomas A. C. Dayton M. R. Stevens-Lapsley J. E. (2014). Strength and functional deficits in individuals with hip osteoarthritis compared to healthy, older adults. Disabil. Rehabil. 36, 307–312. 10.3109/09638288.2013.790491 23659184 PMC4165337

[B29] Jun H.-G. Chang Y.-Y. Dan B.-J. Jo B.-R. Min B.-H. Yang H.. (2011). Walking and sit-to-stand support system for elderly and disabled, in 2011 IEEE International Conference on Rehabilitation Robotics (Zurich: IEEE), 1–5. 10.1109/ICORR.2011.5975365 22275569

[B30] Kawaguchi J. K. Babcock G. (2010). Validity and reliability of handheld dynametric strength assessment of hip extensor and abductor muscles. Athlet. Train. Sports Health Care 2, 11–17. 10.3928/19425864-20101221-04

[B31] Kegelmeyer D. A. Parthasarathy S. Kostyk S. K. White S. E. Kloos A. D. (2013). Assistive devices alter gait patterns in Parkinson disease: advantages of the four-wheeled walker. Gait Posture 38, 20–24. 10.1016/j.gaitpost.2012.10.027 23237981

[B32] Lacey G. J. Rodriguez-Losada D. (2008). The evolution of guido. IEEE Robot. Autom. Magaz. 15, 75–83. 10.1109/MRA.2008.92992427295638

[B33] Lindemann U. Schwenk M. Klenk J. Kessler M. Weyrich M. Kurz F.. (2016). Problems of older persons using a wheeled walker. Aging Clin. Exp. Res. 28, 215–220. 10.1007/s40520-015-0410-8 26226859

[B34] Lindemann U. Schwenk M. Schmitt S. Weyrich M. Schlicht W. Becker C. (2017). Effect of uphill and downhill walking on walking performance in geriatric patients using a wheeled walker. Z. Gerontol. Geriatr. 50, 483–487. 10.1007/s00391-016-1156-4 27878412

[B35] Martins M. Santos C. Frizera A. Ceres R. (2015). A review of the functionalities of smart walkers. Med. Eng. Phys. 37, 917–928. 10.1016/j.medengphy.2015.07.006 26307456

[B36] Mikolajczyk T. Ciobanu I. Badea D. I. Iliescu A. Pizzamiglio S. Schauer T.. (2018). Advanced technology for gait rehabilitation: an overview. Adv. Mech. Eng. 10:1687814018783627. 10.1177/1687814018783627

[B37] Mintken P. E. Carpenter K. J. Eckhoff D. Kohrt W. M. Stevens J. E. (2007). Early neuromuscular electrical stimulation to optimize quadriceps muscle function following total knee arthroplasty: a case report. J. Orthop. Sports Phys. Ther. 37, 364–371. 10.2519/jospt.2007.2541 17710905

[B38] Mitzner T. L. Chen T. L. Kemp C. C. Rogers W. A. (2014). Identifying the potential for robotics to assist older adults in different living environments. Int. J. Soc. Robot. 6, 213–227. 10.1007/s12369-013-0218-7 24729800 PMC3979567

[B39] Moreira R. Alves J. Matias A. Santos C. (2019). Smart and assistive walker–asbgo: rehabilitation robotics: a smart–walker to assist ataxic patients. Adv. Exp. Med. Biol. 1170, 37–68. 10.1007/978-3-030-24230-5_2 32067202

[B40] Mou W.-H. Chang M.-F. Liao C.-K. Hsu Y.-H. Tseng S.-H. Fu L.-C. (2012). Context-aware assisted interactive robotic walker for parkinson's disease patients, in 2012 IEEE/RSJ International Conference on Intelligent Robots and Systems (Vilamoura-Algarve: IEEE), 329–334.

[B41] Mrozowski J. Awrejcewicz J. Bamberski P. (2007). Analysis of stability of the human gait. J. Theor. Appl. Mech. 45, 91–98. Available online at: http://www.ptmts.org.pl/jtam/index.php/jtam/article/view/v45n1p91

[B42] Mundt M. Batista J. P. Markert B. Bollheimer C. Laurentius T. (2019). Walking with rollator: a systematic review of gait parameters in older persons. Eur. Rev. Aging Phys. Act. 16, 1–9. 10.1186/s11556-019-0222-5 31528238 PMC6734589

[B43] Neto A. F. Elias A. Cifuentes C. Rodriguez C. Bastos T. Carelli R. (2015). Smart walkers: Advanced robotic human walking-aid systems, in Intelligent Assistive Robots (Cham: Springer), 103–131.

[B44] Nieuwboer A. Dom R. De Weerdt W. Desloovere K. Fieuws S. Broens-Kaucsik E. (2001). Abnormalities of the spatiotemporal characteristics of gait at the onset of freezing in parkinson's disease. Mov. Disord. 16, 1066–1075. 10.1002/mds.1206 11748737

[B45] NIH Consensus Development Panel on Osteoporosis Prevention, Diagnosis, and Therapy. (2001). Osteoporosis prevention, diagnosis, and therapy. JAMA 285, 785–795. 10.1001/jama.285.6.785 11176917

[B46] Papageorgiou X. S. Chalvatzaki G. Lianos K.-N. Werner C. Hauer K. Tzafestas C. S.. (2016). Experimental validation of human pathological gait analysis for an assisted living intelligent robotic walker, in 2016 6th IEEE International Conference on Biomedical Robotics and Biomechatronics (BioRob) (Singapore: IEEE), 1086–1091.

[B47] Pedersen M. M. Holt N. E. Grande L. Kurlinski L. A. Beauchamp M. K. Kiely D. K.. (2014). Mild cognitive impairment status and mobility performance: an analysis from the boston rise study. J. Gerontol. Ser. A Biomed. Sci. Med. Sci. 69, 1511–1518. 10.1093/gerona/glu063 24799356 PMC4296116

[B48] Pirker W. Katzenschlager R. (2017). Gait disorders in adults and the elderly. Wiener Klinisch. Wochensch. 129, 81–95. 10.1007/s00508-016-1096-4 27770207 PMC5318488

[B49] Poewe W. Seppi K. Tanner C. M. Halliday G. M. Brundin P. Volkmann J.. (2017). Parkinson disease. Nat. Rev. Dis. Primers 3, 1–21. 10.1038/nrdp.2017.13 28332488

[B50] Rikli R. E. Jones C. J. (2013). Senior Fitness Test Manual. Human kinetics.

[B51] Rydevik K. Fernandes L. Nordsletten L. Risberg M. A. (2010). Functioning and disability in patients with hip osteoarthritis with mild to moderate pain. J. Orthop. Sports Phys. Ther. 40, 616–624. 10.2519/jospt.2010.3346 20811166

[B52] Sammer G. Uhlmann T. Unbehaun W. Millonig A. Mandl B. Dangschat J.. (2012). Identification of mobility-impaired persons and analysis of their travel behavior and needs. Transport. Res. Rec. 2320, 46–54. 10.3141/2320-06

[B53] Scheidegger W. M. de Mello R. C. Sierra M. S. D. Jimenez M. F. Munera M. C. Cifuentes C. A.. (2019). A novel multimodal cognitive interaction for walker-assisted rehabilitation therapies, in 2019 IEEE 16th International Conference on Rehabilitation Robotics (ICORR) (Toronto: IEEE), 905–910. 10.1109/ICORR.2019.8779469 31374745

[B54] Sierra M. S. D. Arciniegas-Mayag L. Bautista M. Pinto-Bernal M. J. Cespedes N. Múnera M.. (2022a). Introduction to Robotics for Gait Assistance and Rehabilitation, 1st Edn, Vol. 1. Cham: Springer International Publishing.

[B55] Sierra M. S. D. Garzón M. Munera M. Cifuentes C. A.. (2019). Human–robot–environment interaction interface for smart walker assisted gait: Agora walker. Sensors 19:2897. 10.3390/s19132897 31262036 PMC6650898

[B56] Sierra M. S. D. Jiménez M. F. Frizera-Neto A. Múnera M. Cifuentes C. A. (2022b). Control Strategies for Human–Robot–Environment Interaction in Assisted Gait With Smart Walkers. Cham: Springer International Publishing.

[B57] Sierra M. S. D. Múnera M. Provot T. Bourgain M. Cifuentes C. A.. (2021). Evaluation of physical interaction during walker-assisted gait with the agora walker: strategies based on virtual mechanical stiffness. Sensors 21:3242. 10.3390/s21093242 34067133 PMC8125083

[B58] Sierra S. D. Molina J. F. Gómez D. A. Múnera M. C. Cifuentes C. A. (2018). Development of an interface for human-robot interaction on a robotic platform for gait assistance: Agora smart walker, in 2018 IEEE Andescon (Santiago de Cali: IEEE), 1–7.

[B59] Skinner J. W. Christou E. A. Hass C. J. (2019). Lower extremity muscle strength and force variability in persons with parkinson disease. J. Neurol. Phys. Ther. 43, 56–62. 10.1097/NPT.0000000000000244 30531387

[B60] Stevens-Lapsley J. E. Balter J. E. Kohrt W. M. Eckhoff D. G. (2010). Quadriceps and hamstrings muscle dysfunction after total knee arthroplasty. Clin. Orthop. Relat. Res. 468, 2460–2468. 10.1007/s11999-009-1219-6 20087703 PMC2919870

[B61] United Nations (2020). World Population Ageing 20200 Highlights: Living Arrangements of Older Persons. United Nations.

[B62] Van der Loos H. M. Reinkensmeyer D. J. Guglielmelli E. (2016). Chapter 64:Rehabilitation and health care robotics, in Springer Handbook of Robotics (Cham: Springer International Publishing), 1685–1728.

[B63] Vaughan C. L. (2003). Theories of bipedal walking: an odyssey. J. Biomech. 36, 513–523. 10.1016/S0021-9290(02)00419-0 12600342

[B64] Walz D. M. Newman J. S. Konin G. P. Ross G. (2010). Epicondylitis: pathogenesis, imaging, and treatment. Radiographics 30, 167–184. 10.1148/rg.301095078 20083592

[B65] Wang T. Dune C. Merlet J.-P. Gorce P. Sacco G. Robert P.. (2014). A new application of smart walker for quantitative analysis of human walking, in European Conference on Computer Vision (Cham: Springer), 464–480.

[B66] Werner C. Geravand M. Korondi P. Z. Peer A. Bauer J. M. Hauer K. (2020). Evaluating the sit-to-stand transfer assistance from a smart walker in older adults with motor impairments. Geriatr. Gerontol. Int. 20, 312–316. 10.1111/ggi.13874 32006458

[B67] World Health Organization (2006). Neurological Disorders: Public Health Challenges. World Health Organization.

[B68] World Health Organization (2015). World Report on Ageing and Health. World Health Organization.

[B69] Wu H.-K. Chen H.-R. Chen W.-Y. Lu C.-F. Tsai M.-W. Yu C.-H. (2020). A novel instrumented walker for individualized visual cue setting for gait training in patients with parkinson's disease. Assist. Technol. 32, 203–213. 10.1080/10400435.2018.1525442 30592441

[B70] Yoon S. H. Jun H. G. Dan B. J. Jo B. R. Min B. H. (2012). Hidden marker position estimation during sit-to-stand with walker, in 2012 Annual International Conference of the IEEE Engineering in Medicine and Biology Society (San Diego: IEEE), 1940–1943. 10.1109/EMBC.2012.6346334 23366295

[B71] Zhang M. Artan N. S. Gu H. Dong Z. Burina Ganatra L. Shermon S.. (2018). Gait study of Parkinson's disease subjects using haptic cues with a motorized walker. Sensors 18:3549. 10.3390/s18103549 30347753 PMC6210411

[B72] Zijlstra A. Mancini M. Lindemann U. Chiari L. Zijlstra W. (2012). Sit-stand and stand-sit transitions in older adults and patients with Parkinson's disease: event detection based on motion sensors versus force plates. J. Neuroeng. Rehabil. 9, 1–10. 10.1186/1743-0003-9-75 23039219 PMC3546014

